# Grouping successive freezing of gait episodes has neutral to detrimental effect on freeze detection and prediction in Parkinson’s disease

**DOI:** 10.1371/journal.pone.0258544

**Published:** 2021-10-12

**Authors:** Scott Pardoel, Gaurav Shalin, Edward D. Lemaire, Jonathan Kofman, Julie Nantel

**Affiliations:** 1 Department of Systems Design Engineering, University of Waterloo, Waterloo, ON, Canada; 2 Faculty of Medicine, University of Ottawa and Ottawa Hospital Research Institute, Ottawa, ON, Canada; 3 School of Human Kinetics, University of Ottawa, Ottawa, ON, Canada; University of Alberta, CANADA

## Abstract

Freezing of gait (FOG) is an intermittent walking disturbance experienced by people with Parkinson’s disease (PD). Wearable FOG identification systems can improve gait and reduce the risk of falling due to FOG by detecting FOG in real-time and providing a cue to reduce freeze duration. However, FOG prediction and prevention is desirable. Datasets used to train machine learning models often generate ground truth FOG labels based on visual observation of specific lower limb movements (event-based definition) or an overall inability to walk effectively (period of gait disruption based definition). FOG definition ambiguity may affect model performance, especially with respect to multiple FOG in rapid succession. This research examined whether merging multiple freezes that occurred in rapid succession could improve FOG detection and prediction model performance. Plantar pressure and lower limb acceleration data were used to extract a feature set and train decision tree ensembles. FOG was labeled using an event-based definition. Additional datasets were then produced by merging FOG that occurred in rapid succession. A merging threshold was introduced where FOG that were separated by less than the merging threshold were merged into one episode. FOG detection and prediction models were trained for merging thresholds of 0, 1, 2, and 3 s. Merging slightly improved FOG detection model performance; however, for the prediction model, merging resulted in slightly later FOG identification and lower precision. FOG prediction models may benefit from using event-based FOG definitions and avoiding merging multiple FOG in rapid succession.

## Introduction

Freezing of gait (FOG) is a walking disturbance experienced by approximately 68% of individuals with advanced Parkinson’s disease (PD) [1, 2]. FOG is a sudden inability to walk, usually of short duration, and appears intermittently [3]. Freezing can lead to loss of balance and falls [4], which are a major concern for individuals with PD [5, 6].

External cues (e.g., rhythmic auditory tones, visual, tactile) can improve PD gait [7, 8] and reduce FOG occurrence [9]. However, continuous cueing can lose effectiveness over time; therefore, cueing should be customizable and applied only when a FOG episode is imminent or in progress [9]. Wearable sensors together with machine-learning models have been utilized for FOG detection to provide such intelligent cueing [9]. This decreased freeze duration and helped freezers resume walking [10–12]. With FOG detection, the freeze event would still occur and thus the risk of falling due to freezing remains a concern. A preferred approach would be to identify an oncoming FOG episode just before the onset and provide a cue to alter the gait pattern to prevent the freeze.

FOG detection and prediction models are frequently set up as supervised machine-learning classifiers [13] that utilize training datasets containing both FOG and non-FOG (i.e., steps without freezing). Therefore, accurate manual labeling of the dataset as FOG or non-FOG is essential. Unfortunately, FOG characteristics can vary considerably between individuals and between FOG episodes for the same individual. As described in [14], FOG can occur with small shuffling steps, trembling in place, or a complete lack of movement (akinetic). Subsequently [15], proposed that FOG be defined as “an episodic inability (lasting seconds) to generate effective stepping…”. The definition proposed by [15], has been used by other researchers [16, 17] and encompasses the FOG types described in [14]. However, the definition relies on subjective judgement of “effective” walking and, even when performed by experts, visual FOG assessment is prone to inter-rater discrepancies, especially between different clinical teams [18]. Despite this, expert assessments likely capture the majority of gait deviations and are sufficient for FOG detection, as evidenced by the good detection performance of the resulting models [11, 17, 19–42]. However, FOG prediction cannot be approached the same way since the period before a freeze cannot be easily identified visually. Instead, FOG prediction ground truth is typically identified by selecting walking data immediately before FOG onset (Pre-FOG). Models are trained to differentiate between this Pre-FOG gait, FOG episode, and normal PD walking [13]. Appropriate ground truth labeling can improve the model training set and allow reproducibility and comparison between different studies.

Table 1 presents various definitions used for FOG ground truth labeling in FOG detection and prediction studies. Key phrases such as “episodic inability to generate effective stepping” [17], or “stop in alternating left-right stepping” [43–45], can be subjective and leave room for ambiguity regarding what is considered an “effective” step. This is especially true when activities other than straight line walking are performed, where normal “alternating left-right stepping” is intentionally disrupted (e.g., changing speed or direction, obstacle avoidance). Ambiguity also occurs for festination and small shuffling steps, which are a common FOG subtype [14] and may not be considered as freezes according to some definitions [16, 46]. Table 1 also presents definitions used in FOG detection and prediction studies that are more specific and encompass multiple FOG subtypes. The definition used by [46] lists different ways a freeze might present (e.g., no foot movement, heel lifting while toes stay on the ground, irregular turning rhythm while the pivot foot stays on the ground [46]), whereas [27, 38, 39, 47] use multiple FOG labels according to different types or severities of FOG instances.

**Table 1:** FOG definitions in FOG detection and prediction studies.

FOG Definitions	Source
“The beginning of a FOG event was detected when the gait pattern (i.e., alternating left–right stepping) was arrested, and the end of FOG was defined as the point in time at which the pattern was resumed” (authors reference [14])	[12]
“…the moment of arrested gait pattern, i.e., stop in alternating left-right stepping, as start of a FOG episode, and the instant when the patient resumed a regular gait pattern as end of FOG”	[43–45]
“…an episodic inability to generate effective stepping” (authors reference [15])	[17]
“… an unintentional and temporary phenomenon where the feet failed to progress” (authors reference [14, 15, 48])	[16]
“… an absolute cessation or marked reduction of forward progression of the feet despite the intention to walk” (authors reference [3])	[29]
“… paroxysmal interruption of stride or marked reduction in forward feet progression”	[36]
“… an epoch of time in which patients suddenly became unable to make a turn inside a taped 1 m^2^ box on the floor, despite the intention to do so” (authors reference [29])	[37]
“…when the gait pattern (alternating right and left steps) was arrested or if it appeared as if they were trying unsuccessfully to initiate or continue locomotion/turn. The end of an episode was defined as the time when an effective step had been performed and followed by continuous locomotion.”	[49]
**Definitions including subtypes**	
“(1) slight modification of the gait with no falling risk (green); (2) main gait modification with falling risk (orange); (3) FOG gait is blocked with or without festination (red).”	[38, 39]
“… an intention to walk without movement of the feet, or as heel lifting while toes stay on the ground, or an irregular turning rhythm while the pivot foot stays on the ground” (authors reference [14, 17, 50])	[46]
“… each stride is classified at the output as one of the six types: normal, short^+^ (similar to, but shorter than ‘normal’ strides), short^-^ (very short forward movements, up to 20 cm, with frequencies of the movement in the low (locomotor) band), FOG^+^ (FOG with knee trembling/tremor), FOG^-^ (FOG with complete motor block), and progressive shortening of stride while turning (PST).” *	[47]
No definition provided, however, a distinction is made between trembling in place and shuffling forward FOG subtypes.	[27]

* Locomotor band refers to the 0–3 Hz frequency range.

The definitions in Table 1 can be broadly grouped as event-based [12, 16, 43–47] or periods of gait disruption [17, 29, 36–39, 49]. The event-based definitions focus on specific behaviors of the limbs, such as cessation of foot advancement [16] or failure of the stepping foot to leave the ground [46]. Event-based definitions have a very specific onset (e.g., foot fails to leave the ground) and termination (e.g., foot leaves the ground); however, shuffling FOG or multiple consecutive FOG episodes separated by a few steps would be labeled as many separate freezes, that may be more appropriately classified as a single FOG episode. In contrast, the “periods of gait disruption” definitions are more general and relate to functional locomotion. For example, cessation of “effective stepping” [17] does not specify exact onset and termination timing. Accordingly, shuffling FOG and multiple FOG episodes in quick succession could be considered as a single period of disrupted gait.

In FOG detection and prediction studies, FOG episodes are labeled and datasets are subjected to various assumptions (e.g., ignoring short FOG [32, 46]) and pre-processing steps (e.g., merging FOG episodes [46] or window homogeneity requirements [51]) to refine which frames or data windows are considered as FOG. Since very short duration FOG can be difficult to detect using automatic systems [17] or could be considered a minor gait disturbance, some researchers exclude short FOG from datasets [32, 46]. In [46], FOG shorter than 1 s in duration were labeled as non-FOG gait. Similarly, in [32], only FOG episodes longer than 3 s were considered to be clinically important. In addition to explicitly eliminating FOG episodes based on a duration, short FOG can also be excluded by using a low temporal resolution (e.g., labels applied at one second intervals or longer [24, 52]). Similarly, some FOG episodes can be excluded through windowing, which is the segmentation of walking data into time windows that are used for feature extraction and classification [13]. If the windows are required to be homogeneous (i.e., composed entirely of data with the same label) then all FOG episodes shorter than the chosen window duration are excluded. In many cases, the chosen window length is a compromise between being short enough to capture brief FOG episodes and long enough for specific feature calculations, such as the Freeze Index (FI) [13, 53].

Excluding short FOG may overlook periods of multiple FOG in rapid succession. For example, a person may freeze, take a few ineffective steps while attempting to resume normal walking, then freeze again. According to an event-based FOG definition, multiple FOG episodes in quick succession would be labeled as individual FOG episodes with a few steps in between. If a low temporal resolution for labeling is used (i.e. labels applied at long time intervals), a minimum FOG duration is imposed, or windows are required to be homogeneous, entire sequences of short FOG episodes may be excluded or labeled as normal gait. However, multiple short FOG episodes may be a relevant gait disturbance that should be detected and considered in a cueing system. A FOG definition based on a period of gait disruption would consider a sequence of multiple short FOG episodes as a single FOG occurrence. Combining many short FOG episodes into one FOG occurrence would be less likely to result in discarded data due to windowing or the labeling interval.

Various approaches can be used to merge multiple FOG episodes that occur in quick succession. In [46], FOG episodes separated by less than 1 s were merged. In [51], windows were considered to be FOG if they contained at least 50% FOG data; therefore, as the window moved through the data, two FOG episodes separated by a short non-FOG period, such as one or two small steps, could result in the windows all being labeled as FOG. In [49], the FOG detection model outputs were merged if the model detected FOG separated by less than 2 s. This merging allowed better comparison between the model output and the dataset labels, since labels were generated using a period of gait disruption definition [49].

Currently, evidence is lacking to support the decision to use an “event-based” or “period of gait disruption” approach for classifying FOG. For example, merging in [49] was done to improve the agreement between the model output and expert generated data labels, not due to established recommendations in existing literature. Considering this gap in the literature, the effect of using event-based and period of gait disruption approaches for FOG identification should be examined. The current research determined the effect of merging successive FOG on freeze detection and prediction in PD. The study outcomes can help guide development of objective and appropriate classification models for wearable FOG mitigation systems.

## Methods

### Data collection

Walking data were collected from eleven males with PD, during a single visit to the Movement Performance Laboratory at the University of Ottawa. To be eligible for the study, participants were required to be able to walk unassisted, experience freezing at least once a week, and not have undergone deep brain stimulation therapy or have conditions other than PD that impair gait and balance. Ethics approval was obtained from the University of Ottawa (H-05-19-3547) and University of Waterloo (40954) and all participants provided informed written consent. Participant demographics and questionnaire outcomes are included in Table 2. Participants were on their normal antiparkinsonian medication dosage and schedule. Data collection was generally scheduled such that participants were tested just prior to their next regular medication dose.

**Table 2:** Participant information and questionnaire outcomes.

Participant	Age (years)	Years since diagnosis	NFOG-Q	UPDRS III
**P01**	67	16	14	10
**P02**	80	11	21	20
**P03**	71	11	17	13
**P04**	64	10	4	18
**P05**	70	14	20	13
**P06**	68	19	22	29
**P07**	78	5	15	16
**P08**	70	12	17	20
**P09**	80	10	18	18
**P10**	80	2	4	15
**P11**	72	5	19	20
**Mean**	72.7	10.5	15.5	17.5
**(SD)**	(5.5)	(4.8)	(5.9)	(4.8)

NFOG-Q, New Freezing of Gait Questionnaire; UPDRS-III, Unified Parkinson’s Disease Rating Scale Section III.

**Fig 1:**
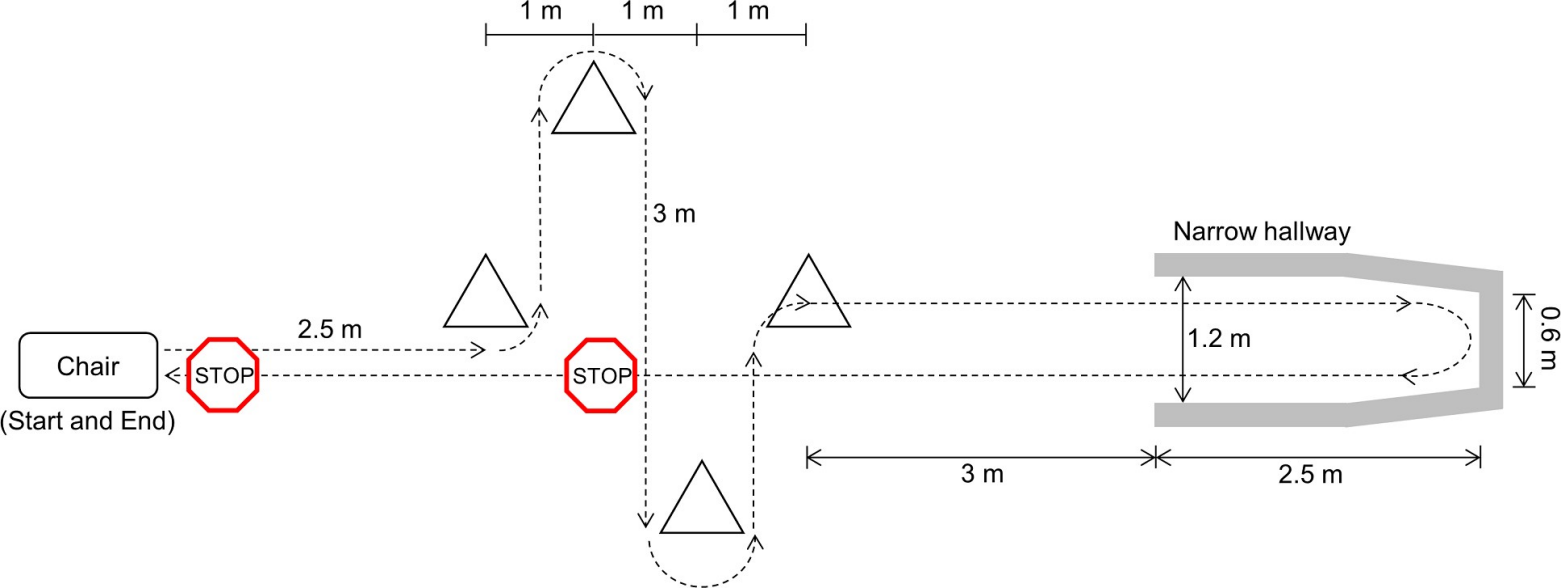
Experiment walking path. Image adapted from [54].

During the lab visit, participants walked a complex path consisting of 90° and 180° turns, stops, starts, and a narrow passageway leading to a dead end (Fig 1). The first stop, during straight-line walking back to the chair, was required to be within the 3 m region delimited by the cones. The stopping location was chosen by the participant and could therefore be different for each trial. For the second stop, participants stopped directly in front of the chair. While walking the path (up to 30 times), participants performed additional physical and verbal tasks to increase the likelihood of freezing. The physical task began with a plastic tray with a small pyramid of 3 wooden blocks on top. To increase difficulty, if necessary, the blocks were replaced with a paper cup or the tray was held with one hand while a sealed water bottle was held in the other hand. The verbal task consisted of saying as many words as possible beginning with a specific letter. The words were required to be different, and proper nouns or very similar words were not allowed. A total of 241 minutes of walking data were collected, during which seven participants froze 362 times.

**Fig 2:**
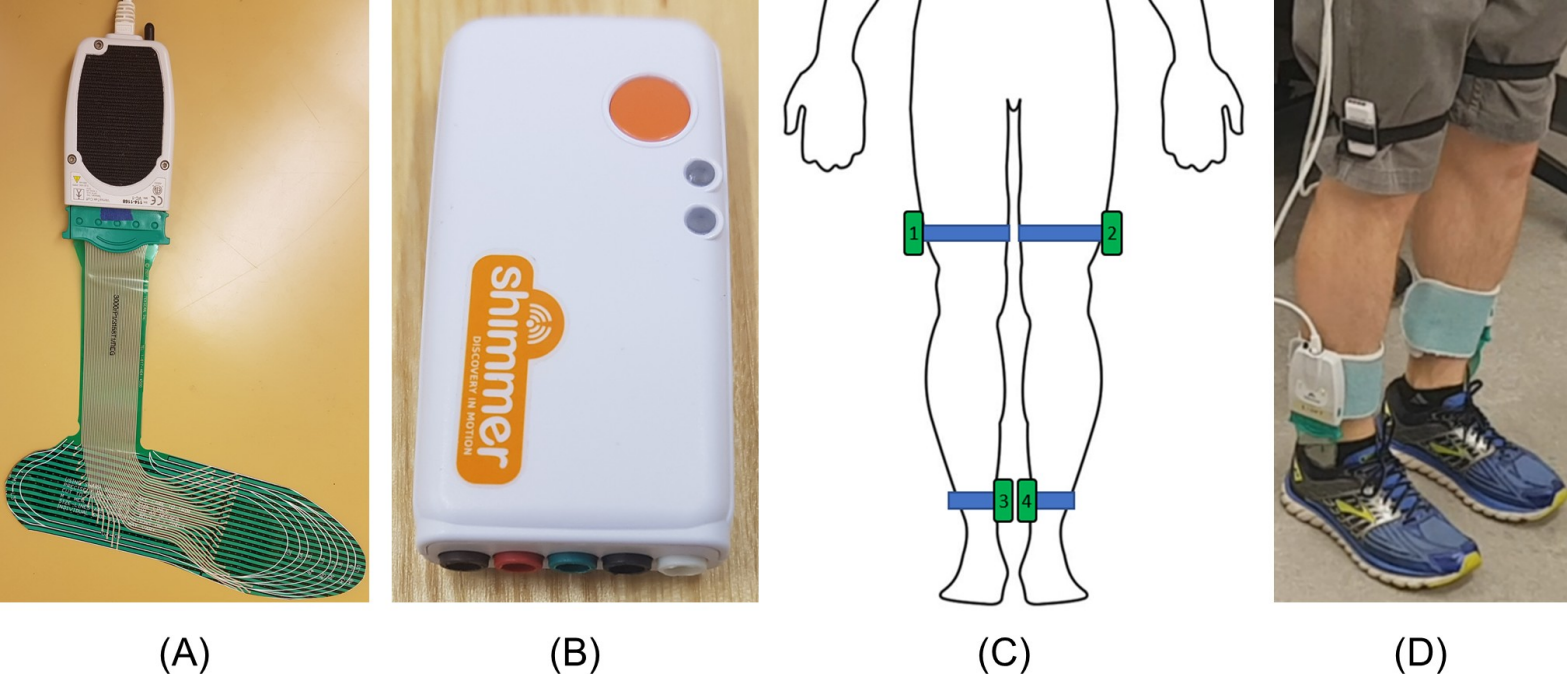
Sensors systems used in data collection. (A) FScan pressure sensing insole. (B) Shimmer3 IMU sensor. (C) Diagram of IMU placement. (D) Insole and IMU systems on body. Modified from [54].

FScan pressure sensing shoe insoles (Tekscan, Boston, MA) and Shimmer3 inertial measurement units (IMU) (Shimmer, Ireland) were used to collect walking data (Fig 2). FScan sensors are thin (less than 1 mm), flexible, insoles with 3.9 pressure sensing cells per cm^2^ (Fig 2A). Prior to participant arrival, a new pair of insoles was equilibrated using a pressurized air bladder. The insoles were then cut to fit inside the participant’s regular shoes. Prior to beginning the trials, a step calibration was performed, during which the participant shifted all of their weight from one foot to the other and back again. The plantar pressure data were collected at 100 Hz.

Four IMU sensors were used to collect acceleration (± 4 g) and gyroscope (± 500 dps) data with a sampling rate of 512 Hz. The sensors were positioned on the lateral thigh just above the knees and on the medial shank just above the ankle and held with elastic straps. Acceleration and gyroscope data were downsampled to 100 Hz to match the plantar pressure sampling rate. Walking trials were video recorded using a smartphone camera for post collection FOG identification. Each trial began with a single stomp of the right foot, which was used to synchronize the video, plantar pressure, and IMU data. The synchronization was confirmed during the labeling process by examining multiple heel-strike events.

### FOG definition and merging approach

The collected data were synchronized, visually inspected, and labeled using a custom labeling program written with MATLAB R2019b App Designer. All data processing and model development were performed in the MATLAB environment (MathWorks, MA, USA). During data collection, authors SP and JN identified FOG occurrences. In post processing, SP identified the onset and termination of FOG episodes to a resolution of 30 Hz. In case of uncertainty, the second rater was consulted. The beginning of a freeze was defined as “the instant the stepping foot fails to leave the ground despite the clear intention to step”. The end of the freeze was defined as “the instant the stepping foot begins or resumes an effective step”. For example, a step was considered effective the instant the heel lifted from the ground, provided that it was followed by a smooth toe off with the entire foot lifting from the ground and advancing into the next step without loss of balance. As a special case if a person froze, stopped trying to advance, and remained standing, the instant that the participant stopped trying to advance was considered the end of the freeze. This was determined by the complete absence of foot movement and known FOG characteristics such as trembling of the knee, medial-lateral weight shifting, or attempt at shuffling.

To determine the effect of merging successive freeze episodes on freeze detection and prediction, consecutive freezes were merged into a single freeze if the time between the beginning of a FOG episode and the end of the previous FOG episode was less than a merging threshold. All data between the two freezes were relabeled as FOG, thus forming a single longer FOG episode. Merging thresholds of 0, 1, 2, and 3 s were tested.

### FOG prediction and detection models

**Fig 3:**
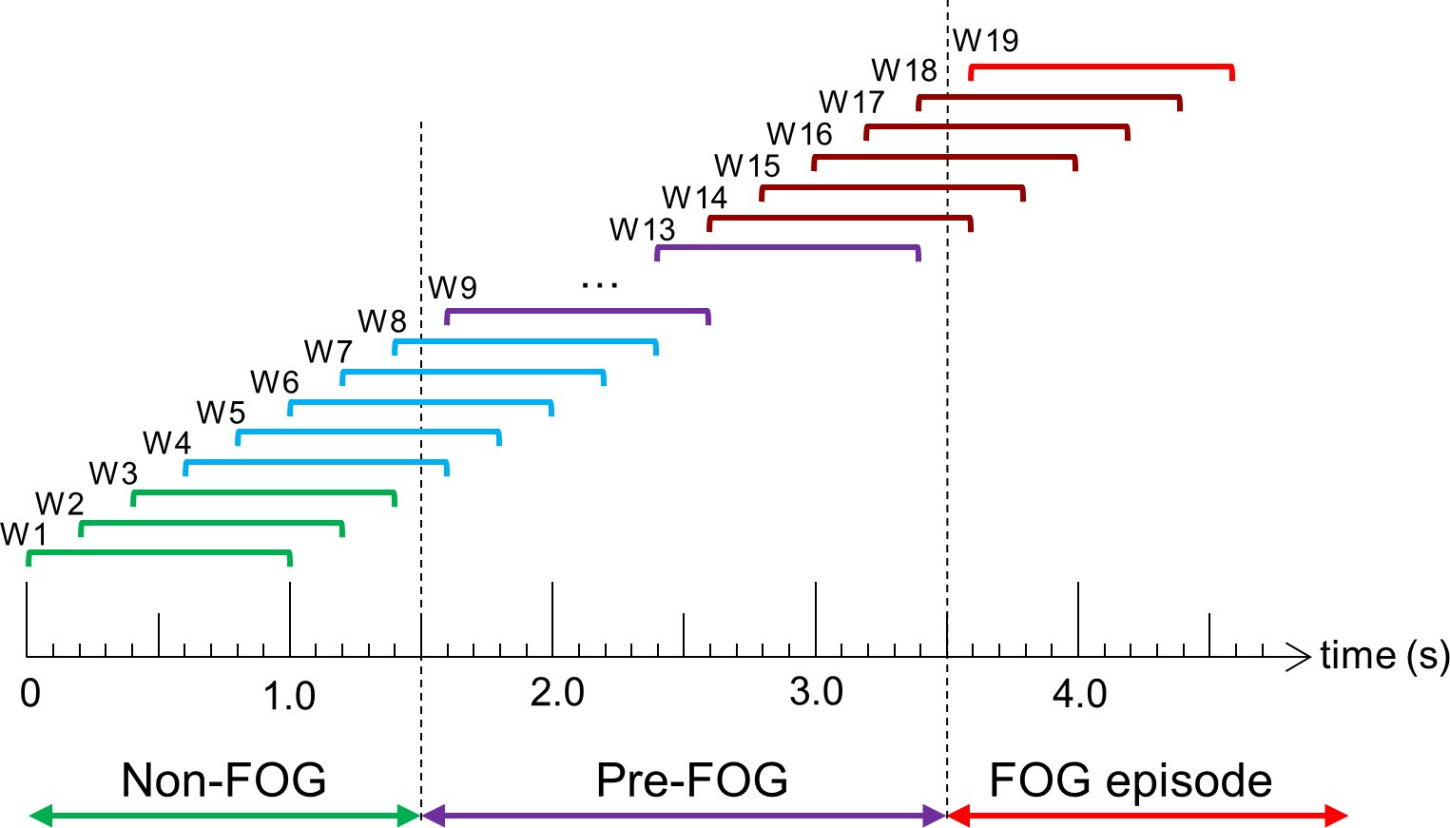
Diagram of windowing approach. Windows W1-W3 contain only non-FOG data, W4-W8 contain both non-FOG and Pre-FOG data, W9-W13 contain only Pre-FOG data, W14-W18 contain both Pre-FOG and FOG data, and W19 contains only FOG data.

Data were labeled as Non-FOG, Pre-FOG, or FOG. Pre-FOG was defined as 2 s of data before each FOG, as in [54, 55]. The labeled data were divided into 1 s windows with 0.2 s shift between windows (i.e., 80% overlap) (Fig 3). Similar to [51], the windows were not required to be homogeneous; therefore, windows could contain different labels, which ensured that no FOG data were discarded.

For detection models, the target class included all windows that contained any FOG data. For example, in Fig 3 windows (W) containing FOG and Pre-FOG (W14-W18) and purely FOG (W19), as well as windows containing FOG and non-FOG data (not shown) were included in the target class. The non-target class contained all other data.

For prediction models, the target class contained the windows beginning anytime during the 2 seconds prior to FOG onset (W9-W18). This included windows beginning and ending during the 2 seconds prior to FOG onset (W9-W13) as well as windows that include some Pre-FOG and some FOG data (W14-W18). Windows that contained only non-FOG data (W1-W3), non-FOG and Pre-FOG (W4-W8), and only FOG data (W19) were in the non-target class for FOG prediction models (Fig 3).

Ten features were extracted from each window. The features were calculated from lower-limb IMU and plantar pressure data and were selected by Relief-F feature ranking from among over 850 total features, described in [54]. The features used were the dominant fast Fourier transform (FFT) frequency of foot centre of pressure (COP) velocity in medial/lateral (ML) directions for the right leg and anterior/posterior (AP) for the right and left legs, the dominant FFT frequency of thigh accelerometers in the AP direction for the right and left legs, mean energy of wavelet transform (WT) approximation coefficient of COP position in the AP direction for the right leg, number of COP AP reversals for the right and left legs, mean of WT approximation coefficient for COP position in AP for the right leg, and the min of the COP detail coefficient in the AP direction for the right leg. A two-class decision tree ensemble composed of 100 trees with a maximum of five splits each was trained using random undersampling boosting (RUSBoost). A leave-one-freezer-out cross validation was performed for all models. In leave-one-freezer-out cross validation, model training uses data from all but one participant who froze and model testing uses data from the remaining participant. The process is repeated for each freezer and the model performance results are averaged across all iterations. To examine the effect of FOG merging on FOG detection performance, the decision tree ensemble was trained repeatedly using identical model parameters but with different merging thresholds (0, 1, 2, 3 s). The datasets created with separate freeze episodes and with merged freeze episodes using different merging thresholds have been made publicly available [56].

### FOG prediction and detection model evaluation

**Fig 4:**
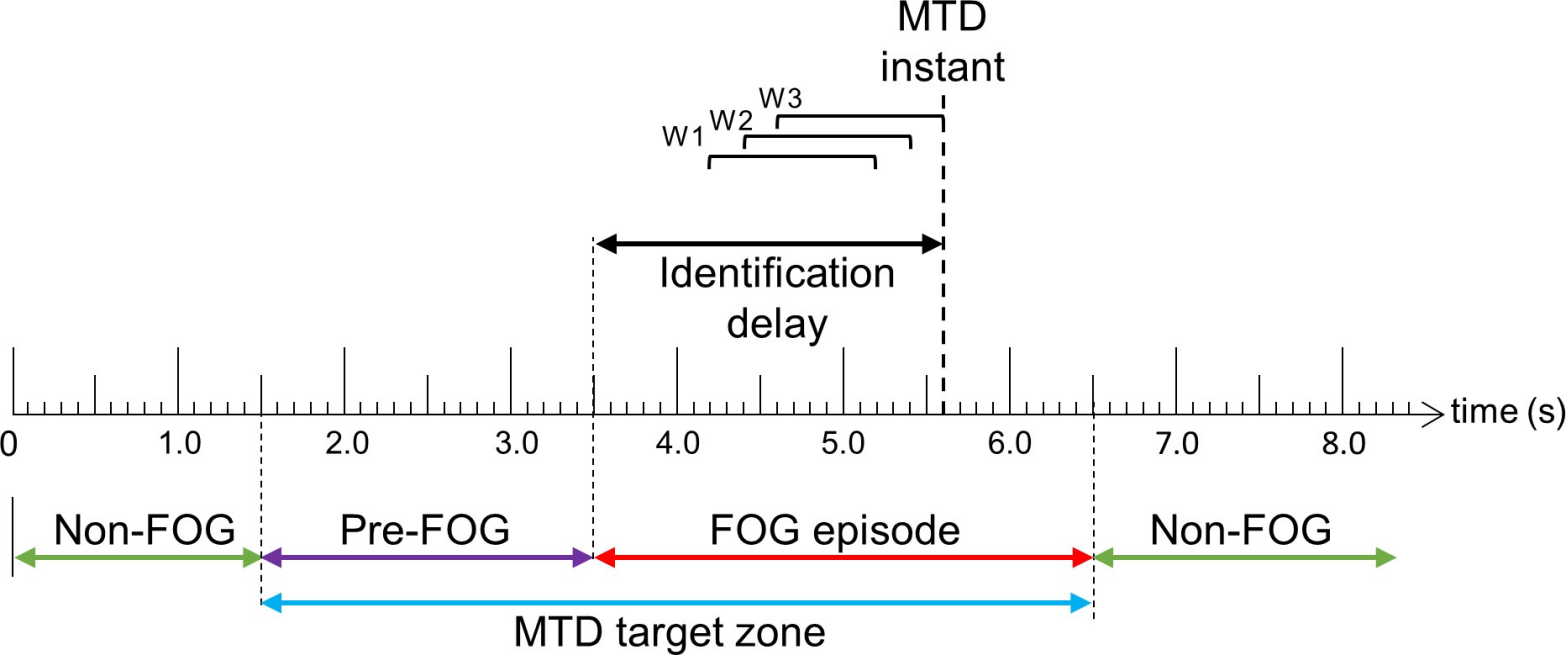
Diagram of FOG episode identification using the FOG episode-based evaluation. Three consecutive positive window classifications (W1-W3) result in a model trigger decision (MTD) at the end of the third window (MTD instant). To be correctly identified, a FOG episode requires the MTD instant to be within the MTD target zone. Identification delay is the time difference between FOG onset and the MTD.

The trained models were evaluated using windows and FOG episodes. The window-based evaluation compared each window classification to the ground truth label and calculated sensitivity and specificity. While sensitivity and specificity are useful measures, this evaluation does not necessarily reflect a model’s ability to act as a timely trigger for a cueing system since a model may only detect freeze windows and trigger a cue at the end of a FOG episode. Therefore, the FOG-episode-based evaluation determined if and when each episode was detected by the model. To avoid cues caused by misclassified windows, three consecutive positive target class classifications were required to generate a model trigger decision (MTD) (i.e., three previous windows had to be classified as belonging to the target class, Fig 4). For each FOG episode in the test data, a MTD target zone was defined as the period that includes the Pre-FOG data and FOG episode (2 s prior to FOG onset until the end of the FOG episode) (Fig 4), since a cue within this target zone would be helpful to either prevent or mitigate a FOG episode. The episode was considered to be correctly identified if at least one MTD occurred within the MTD target zone. For each correct FOG episode identification, the identification delay (ID) was calculated as the time difference between the FOG onset and the MTD. If a FOG episode resulted in multiple MTD, then the earliest MTD within the target zone was used to determine the identification delay for that episode. Positive FOG ID values indicated that the MTD occurred after FOG onset, and negative FOG ID values indicated that the MTD occurred before FOG episode onset.

The number of MTD true positives (MTD within MTD target zone) and false positives (MTD outside the zone) were determined and used to calculate model precision. Lastly, a hypothetical cueing protocol was introduced to demonstrate how the system might be used in a real cueing application. Since cueing is intended to modify the person’s gait, a period is needed to allow the gait to change before another cue would be given. Therefore, a 2.5 s no-cue interval, during which all MTD would be ignored, was used. If a FOG episode began within a no-cue interval, then this freeze was considered to have been identified, since it follows a MTD.

## Results

Table 3 presents the number of FOG episodes experienced by each participant for different merging thresholds. Merging FOG episodes reduced the number of FOG episodes, primarily for Participants P07 and P08.

**Table 3:** Number of FOG episodes for each participant for different merging thresholds.

Participant	Number of FOG episodes				Reduction in number of episodes by merging with *mt* = 3 s
	*mt* = 0 s	*mt* = 1 s	*mt* = 2 s	*mt* = 3 s	
**P01**	49	48	48	48	1
**P02**	35	35	35	35	0
**P03**	14	14	13	13	1
**P04**	0	0	0	0	-
**P05**	0	0	0	0	-
**P06**	10	10	10	10	0
**P07**	221	171	118	87	134
**P08**	24	16	14	14	10
**P09**	9	9	9	7	2
**P10**	0	0	0	0	-
**P11**	0	0	0	0	-

*mt*, merging threshold.

For window-based FOG detection (Table 4), sensitivity and specificity averages across all participants changed little (≤ ± 1%) due to merging (mean sensitivity: 83.4% for *mt* = 2 s, compared to 82.4% for *mt* = 0 s; mean specificity: 87.9% for *mt* = 2,3 s, compared to 88.3% for *mt* = 0 s). This included Participants P07 and P08, who had the largest reduction in number of FOG episodes due to merging (Table 3). For the prediction models (Table 5), mean sensitivity decreased slightly as the merging threshold increased (68.4% for *mt* = 2 s, from 73.4% for *mt* = 0 s). Mean specificity was highest (82.8%) for *mt* = 2 s and lowest (80.9%) for *mt* = 3 s.

**Table 4:** Window-based FOG detection model performance for various merging thresholds.

Participant	*mt* = 0 s		*mt* = 1 s		*mt* = 2 s		*mt* = 3 s	
	Sens	Spec	Sens	Spec	Sens	Spec	Sens	Spec
	(%)	(%)	(%)	(%)	(%)	(%)	(%)	(%)
**P01**	88.1	88.2	88.3	88.3	89.5	87.3	88.8	87.4
**P02**	81.0	90.2	81.4	90.4	80.6	90.1	81.0	90.2
**P03**	70.6	93.1	72.0	93.0	74.8	93.1	73.4	93.0
**P06**	90.6	90.7	93.8	90.3	90.6	90.6	93.8	90.3
**P07**	64.9	86.9	65.1	86.4	63.2	86.6	61.3	86.8
**P08**	87.2	87.2	87.2	87.2	86.6	87.3	87.0	87.0
**P09**	94.4	81.6	93.1	80.8	98.6	80.4	94.1	80.4
**Mean**	82.4	88.3	83.0	88.1	83.4	87.9	82.8	87.9
**(SD)**	(10.1)	(3.4)	(10.0)	(3.6)	(10.8)	(3.7)	(11.0)	(3.7)

*mt*, merging threshold; Sens, sensitivity; Spec, specificity.

**Table 5:** Window-based FOG prediction model performance for various merging thresholds.

Participant	*mt* = 0 s		*mt* = 1 s		*mt* = 2 s		*mt* = 3 s	
	Sens (%)	Spec (%)	Sens (%)	Spec (%)	Sens (%)	Spec (%)	Sens (%)	Spec (%)
**P01**	70.5	81.7	68.8	80.6	63.1	82.0	67.3	79.0
**P02**	55.3	83.5	57.3	83.1	58.2	83.4	59.0	82.3
**P03**	61.7	93.4	60.2	93.9	57.4	94.3	63.1	93.1
**P06**	87.1	88.7	85.1	89.1	82.2	90.7	82.2	90.3
**P07**	72.2	67.5	68.6	67.0	66.2	66.7	65.6	64.7
**P08**	77.6	84.3	71.2	84.5	69.4	85.6	67.2	82.8
**P09**	89.2	75.6	87.8	76.1	82.4	76.7	78.6	73.7
**Mean**	73.4	82.1	71.3	82.1	68.4	82.8	69.0	80.9
**(SD)**	(11.5)	(7.9)	(10.7)	(8.1)	(9.6)	(8.4)	(7.7)	(9.0)

*mt*, merging threshold; Sens, sensitivity; Spec, specificity.

Results for the FOG episode-based evaluation are presented in Tables 6 and 7. For the FOG detection model (Table 6), the mean percentage of correctly identified FOG episodes increased from 91.3% for 0 s merging threshold to 93.3% for 2 s merging threshold. For the prediction model (Table 7), the mean percentage of correctly identified FOG episodes increased from 94.0% (0 s threshold) to 95.9% (3 s threshold). For the detection model, the highest percentage of correctly identified FOG episodes occurred using a 2 s merging threshold. For prediction, the highest percentage was achieved with a 3 s merging threshold.

**Table 6:** Episode-based FOG detection model performance for various merging thresholds.

Participant	*mt* = 0 s		*mt* = 1 s		*mt* = 2 s		*mt* = 3 s	
	Episodes identified	Average ID	Episodes identified	Average ID	Episodes identified	Average ID	Episodes identified	Average ID
	(%)	(s)	(%)	(s)	(%)	(s)	(%)	(s)
**P01**	91.8	0.02	91.7	0.04	93.8	0.01	93.8	0.03
**P02**	85.7	0.48	85.7	0.49	85.7	0.47	85.7	0.47
**P03**	71.4	-0.34	71.4	-0.32	84.6	-0.13	76.9	-0.18
**P06**	100.0	-0.35	100.0	-0.41	100.0	-0.35	100.0	-0.41
**P07**	90.0	-0.72	90.1	-0.62	89.0	-0.21	88.5	-0.08
**P08**	100.0	-1.09	100.0	-0.73	100.0	-0.53	100.0	-0.56
**P09**	100.0	-0.83	100.0	-0.78	100.0	-1.10	100.0	-0.58
**Mean**	91.3	-0.40	91.3	-0.33	93.3	-0.26	92.1	-0.19
**(SD)**	(9.7)	(0.50)	(9.7)	(0.43)	(6.4)	(0.45)	(8.2)	(0.34)

*mt*, merging threshold; ID, identification delay. Episodes identified are a percentage of the total number of FOG episodes for each participant. Identification delay (ID) indicates the average time between FOG onset and freeze identification (MTD). Positive delay indicates FOG identified after onset, negative delay indicates FOG identified before onset.

**Table 7:** Episode-based FOG prediction model performance for various merging thresholds.

Participant	*mt* = 0 s		*mt* = 1 s		*mt* = 2 s		*mt* = 3 s	
	Episodes identified	Average ID	Episodes identified	Average ID	Episodes identified	Average ID	Episodes identified	Average ID
	(%)	(s)	(%)	(s)	(%)	(s)	(%)	(s)
**P01**	95.9	-0.02	95.8	0.00	89.6	0.04	91.7	-0.01
**P02**	94.3	0.30	94.3	0.27	97.1	0.27	100.0	0.30
**P03**	78.6	-0.33	64.3	-0.49	76.9	-0.26	92.3	-0.28
**P06**	100.0	-0.49	100.0	-0.49	100.0	-0.59	100.0	-0.61
**P07**	97.3	-1.17	97.1	-1.01	95.8	-0.83	94.3	-0.76
**P08**	91.7	-1.15	100.0	-0.81	100.0	-0.72	92.9	-1.08
**P09**	100.0	-1.10	100.0	-1.12	100.0	-0.98	100.0	-0.92
**Mean**	94.0	-0.56	93.1	-0.52	94.2	-0.44	95.9	-0.48
**(SD)**	(6.9)	(0.55)	(11.9)	(0.47)	(7.9)	(0.43)	(3.6)	(0.46)

*mt*, merging threshold; ID, identification delay. Episodes identified are a percentage of the total number of FOG episodes for each participant. Identification delay (ID) indicates the average time between FOG onset and freeze identification (MTD). Positive delay indicates FOG identified after onset, negative delay indicates FOG identified before onset.

For the detection model, changing merging thresholds from 0 s to 3 s, led to FOG identification (earliest MTD) occurring 0.21 s later (changing from -0.4 s to -0.19 s). When changing merging threshold from 0 s to 2 s, which had the best percentage of correctly identified FOG episodes, the mean ID occurred 0.14 s later (-0.4 to -0.26 s). For the prediction model, changing merging thresholds from 0 s to 3 s led to the FOG identification (earliest MTD) occurring 0.08 s later (changing from -0.56 s to -0.48 s). For both detection and prediction models, a negative ID indicated a FOG prediction since the FOG identification was before FOG onset.

The number of true positive (TP) and false positive (FP) MTD for each participant are presented in Tables 8 and 9. The precision of the detection model increased minimally (0.2%) with merging, with 40.3% precision for a 3 s merging threshold. Prediction model precision decreased from 19.4% to 14.3% as the merging threshold increased from 0 s to 3 s.

**Table 8:** MTD precision for the FOG detection model.

Participant	*mt* = 0 s			*mt* = 1 s			*mt* = 2 s			*mt* = 3 s		
	TP	FP	PR	TP	FP	PR	TP	FP	PR	TP	FP	PR
			(%)			(%)			(%)			(%)
**P01**	324	231	58.4	323	227	58.7	330	245	57.4	321	236	57.6
**P02**	436	346	55.8	443	337	56.8	434	346	55.6	436	343	56.0
**P03**	79	268	22.8	82	276	22.9	87	270	24.4	81	271	23.0
**P06**	221	575	27.8	233	608	27.7	221	570	27.9	232	604	27.8
**P07**	1147	409	73.7	1128	437	72.1	1131	407	73.5	1196	414	74.3
**P08**	211	391	35.0	213	381	35.9	206	374	35.5	206	373	35.6
**P09**	62	797	7.2	61	844	6.7	67	851	7.3	71	836	7.8
**Total**	2480	3017		2483	3110		2476	3063		2543	3077	
**Mean**			40.1 (21.6)			40.1 (21.5)			40.2 (21.2)			40.3 (21.5)
**(SD)**												

*mt*, merging threshold; TP, true positive (MTD within MTD target zone); FP, false positive (MTD outside MTD target zone); PR, precision (PR = TP/ (TP+ FP) ×100).

**Table 9:** MTD precision for the FOG prediction model.

Participant	*mt* = 0 s			*mt* = 1 s			*mt* = 2 s			*mt* = 3 s		
	TP	FP	PR	TP	FP	PR	TP	FP	PR	TP	FP	PR
			(%)			(%)			(%)			(%)
**P01**	171	383	30.9	162	377	30.1	137	338	28.8	154	429	26.4
**P02**	117	705	14.2	123	721	14.6	126	699	15.3	132	781	14.5
**P03**	41	205	16.7	40	154	20.6	32	157	16.9	42	213	16.5
**P06**	73	728	9.1	70	649	9.7	63	474	11.7	67	535	11.1
**P07**	998	1565	38.9	793	1641	32.6	508	1691	23.1	365	1863	16.4
**P08**	120	437	21.5	78	413	15.9	64	342	15.8	66	492	11.8
**P09**	48	1008	4.5	46	883	5.0	44	832	5.0	32	1010	3.1
**Total**	1568	5031		1312	4838		974	4533		858	5323	
**Mean**			19.4 (11.2)			18.3 (9.4)			16.7 (7.1)			14.3 (6.5)
**(SD)**												

*mt*, merging threshold; TP, true positive (MTD within MTD target zone); FP, false positive (MTD outside MTD target zone); PR, precision (PR = TP/ (TP+ FP) ×100).

Table 10 presents the results of the hypothetical cueing protocol using a 2.5 s no-cue interval after each triggered cue. The no-cue interval was applied to the detection and prediction models with the highest precision (i.e., the detection model with 3 s merging threshold and prediction model with 0 s merging threshold). For detection model episode identification, the no-cue interval did not change the percent of identified FOG. For the prediction model, the no-cue interval reduced the mean percentage of identified FOG slightly (94% to 93.8%). This decrease was due solely to Participant P07 for whom the percentage of identified FOG episodes decreased from 97.3% to 96.4%. The no-cue interval reduced the number of false positive and true positive MTD for both the detection and prediction models. The no-cue interval decreased mean detection model precision from 40.3% (*mt* = 3 s) to 31.8% and increased mean prediction model precision from 19.4% (*mt* = 0 s) to 30.6%.

**Table 10:** MTD precision for FOG prediction and detection models using a 2.5 s no-cue interval between consecutive cues.

Participant	Detection Model				Prediction model			
	*mt* = 3 s (2.5 s no-cue interval)				*mt* = 0 s (2.5 s no-cue interval)			
	Episodes identified (%)	TP	FP	PR (%)	Episodes identified (%)	TP	FP	PR (%)
**P01**	93.8	45	40	52.9	95.9	47	51	48.0
**P02**	85.7	45	73	38.1	94.3	50	76	39.7
**P03**	76.9	11	67	14.1	78.6	12	54	18.2
**P06**	100.0	23	108	17.6	100.0	24	109	18.0
**P07**	88.5	187	98	65.6	96.4	222	164	57.5
**P08**	100.0	25	67	27.2	91.7	23	64	26.4
**P09**	100.0	10	132	7.0	100.0	11	165	6.3
**Total**		346	585			389	683	
**Mean**	92.1			31.8	93.8			30.6
**(SD)**	(8.2)			(19.9)	(6.8)			(17.0)

*mt*, merging threshold; ID, identification delay; TP, true positive (MTD within MTD target zone); FP, false positive (MTD outside MTD target zone); PR, precision (PR = TP/ (TP+ FP) ×100). Episodes identified are a percentage of the total number of FOG episodes for each participant.

**Fig 5:**
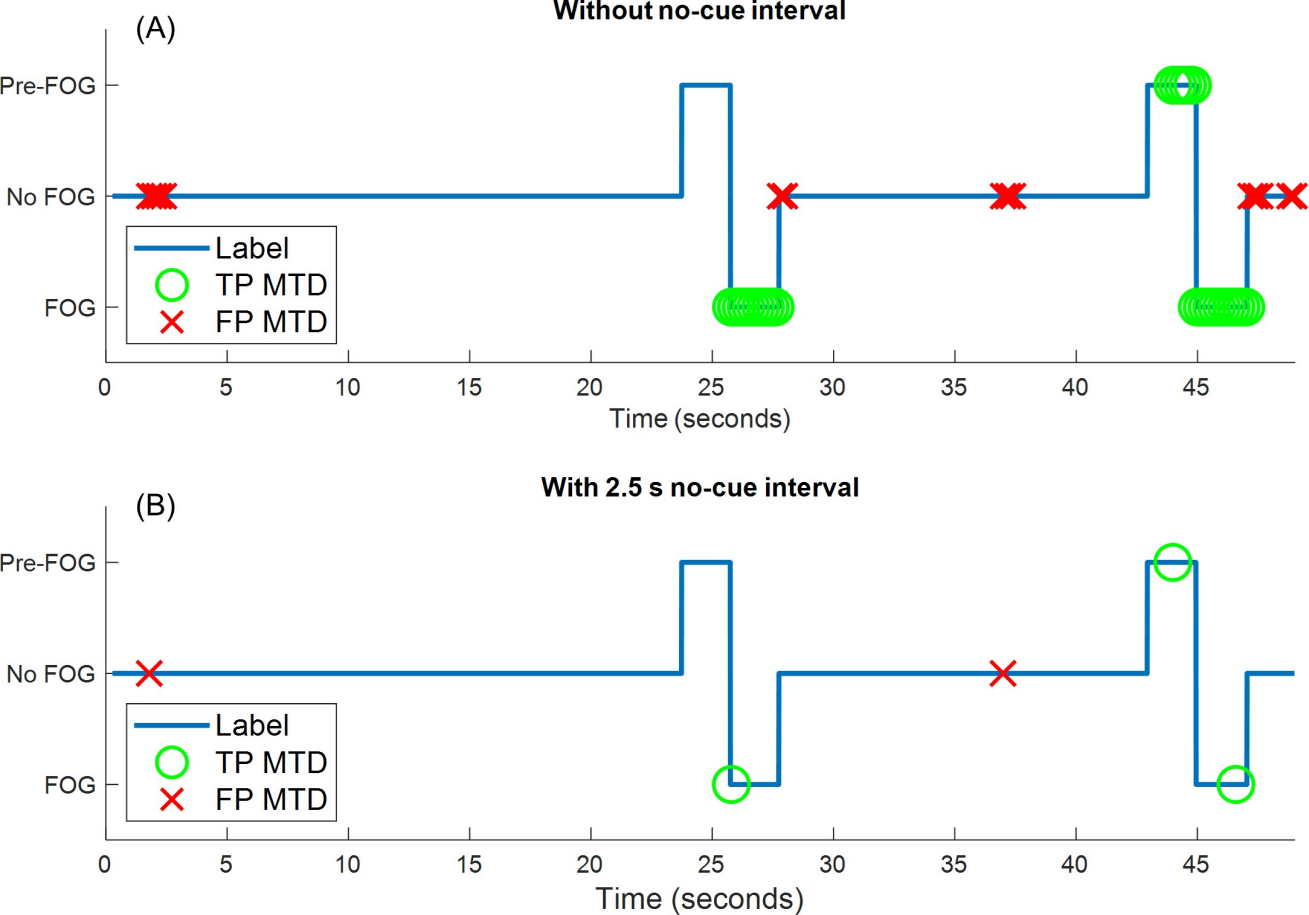
Example session of walking data classification and freeze identification. (A) Without no-cue interval. (B) With 2.5 s no-cue interval. TP MTD: true positive model trigger decision (MTD within MTD target zone), FP MTD: false positive model trigger decision (MTD outside MTD target zone).

Fig 5 shows an example walking session with the MTD TP and FP. Without a no-cue interval (Fig 5A), the first FOG episode was detected at the beginning of the episode (leftmost green circle at approximately 26 s), the second FOG was predicted approximately 1 s before FOG onset (multiple MTD starting at approximately 44 s), and MTD occurred in groups of consecutive windows for both the TP MTD (green) and FP MTD (red). When the no-cue interval was used (Fig 5B), there was also successful FOG identification at the beginning of the episode (TP MTD at 26 s), successful FOG prediction (TP MTD at 44 s), and the number of false positive MTD was reduced from 15 (Fig 5A) to 2 (Fig 5B).

## Discussion

The best performing FOG detection model used a 2 s merging threshold, whereas the best prediction model had a 0 s merging threshold (i.e., no merging). For the window-based evaluation, there was very little difference in model performance for all detection models, across merging thresholds, and a slight difference in performance for prediction models. Model performance was similar to other person-independent FOG detection [32, 46, 51, 57, 58] and prediction [55, 59–61] models in the literature.

For FOG episode-based analysis, the percentage of successfully identified FOG episodes increased slightly due to FOG-episode merging for both the detection (+ 2.0%) and prediction (+ 1.9%) models. The prediction model with a 3 s merging threshold outperformed the detection model by identifying 95.9% of FOG episodes. For all merging thresholds of the detection and prediction models, FOG episodes were identified prior to the FOG onset; therefore, both detection and prediction models were able to predict FOG.

The earliest predictions occurred without merging (0 s merging threshold). Individual participant FOG identification was as early as 1.09 s before FOG onset for the detection model (P08, Table 6), and 1.10 s to 1.17 s before FOG onset for the prediction model (P09-P07, Table 7). When averaged across participants, the earliest identifications were 0.40 s before FOG onset for the detection model and 0.56 s before FOG onset for the prediction model, which both occurred with no merging. The FOG identification was closer to freeze initiation when the merging threshold was 3 s for detection (0.19 s before FOG onset) and 2 s for prediction (0.44 s before FOG onset). Therefore, a merging threshold of 3 s for detection and 2 s for prediction would provide the shortest time for preventative cueing. Merging FOG episodes may not be beneficial in a preventative cueing system since merging led to later FOG identifications but similar FOG identification percentages.

For the detection model, less than ± 0.2% differences in MTD precision were found between merging thresholds. For the prediction model, increasing the merging threshold from 0 s to 3 s decreased the number of true positive MTD from 1568 to 858 and increased the number of false positives from 5031 to 5323, resulting in a 5.1% decrease in precision. This may be the result of having fewer data windows in the target class during training due to merging. Also, there were more FP compared to TP, for both detection and prediction models.

Models tended to produce grouped zones of MTD (Fig 5A), likely because of the 80% overlap between consecutive windows, where data in successive windows are similar and lead to the same classification. To reduce FP, a larger shift between windows may be helpful [46]; however, this would decrease the temporal resolution of a cueing protocol. Using the 2.5 s no-cue interval greatly reduced the number of false positive MTD (5323 to 585 for detection, 5031 to 683 for prediction) by excluding consecutive FP MTD after the first MTD in the group. As shown in Fig 5, a TP MTD near the end of a FOG episode can eliminate FP MTD immediately after the end of the FOG episode, since the FP MTD would fall within the no-cue interval. For the detection model, the no-cue interval had no effect on the percentage of identified FOG episodes. For the prediction model, the no-cue interval only affected the percentage of identified FOG episodes for Participant P07, and this was only a 0.9% difference. P07 had many short FOG in rapid succession. FOG episodes that began within a no-cue interval were considered to be successfully detected, whereas Pre-FOG data for subsequent short freezes within the no-cue interval were ignored. Therefore, models using the no-cue interval may miss FOG episodes that otherwise would have been predicted from the Pre-FOG data. However, these missed episodes do not necessarily indicate decreased model performance, since in a real application, if a cue were given, the subsequent (missed) episode may never occur. In this research, 2.5 s was considered enough time for the person to respond to the cue and for the model to collect addition data that will inform the next classification. Further study is required to determine the time required for gait to adjust following a cue, if the time is person or FOG-subtype specific, and whether subsequent FOG episodes can be avoided. The results could then be used as relevant parameters for personalized FOG cueing systems. For example, the user’s reaction to the cue could be the input of a secondary classifier that is trained using post-cue data. The secondary classifier could determine if the gait parameters are stabilizing and cueing can be stopped, or the gait remains abnormal and cueing should be continued or modulated.

The FOG episode merging results provide guidance for future research. For the FOG detection model, merging successive FOG episodes produced a slight performance improvement. The window-based and FOG episode-based evaluations had their best FOG detection performance when the merging threshold was 2 s, while the highest model precision was for the 3 s merging threshold. However, the non-merged case (0 s merging threshold) resulted in the earliest MTD for the detection models. Thus, the detection model performed better with merging at the cost of identifications being made less in advance, yet still prior to FOG onset. Early detection may not be necessary for non-cueing applications of FOG detection (e.g., gait monitoring [20, 62, 63]), therefore merging FOG episodes may be beneficial.

For the prediction model, FOG episode merging increased the percentage of identified FOG episodes, but slightly decreased window-based sensitivity and specificity, decreased model precision, and resulted in less time between identification and freeze onset. The improvement in percentage of identified FOG episodes was at the cost of identifications being made later. For a FOG prediction model intended to be used in a cueing system, where early detection of FOG may be important, the merging of FOG episodes could be detrimental.

This study utilized data from 11 participants, 7 of whom froze. Future research will aim to integrate models into a real-time system and validate the models on a larger dataset with additional participants. Furthermore, the effect of merging episodes and no-cue intervals for different FOG subtypes and for specific activities, such as turning, could be examined.

## Conclusion

This research examined the effects of defining FOG either as a period of gait disruption (merging successive FOG), or based on an event (no merging), on FOG detection and prediction. For detection, defining FOG as a period of gait disruption produced slightly better results than the event-based definition. Therefore, for FOG detection systems, expert labeling based on periods of ineffective gait is likely sufficient and labeling the onset and termination of each successive FOG episode within a larger period of gait disruption may not be required. However, prediction model performance was adversely affected by increasing the merging threshold, specifically in terms of precision. Therefore, FOG prediction models should be trained using event-based FOG definitions (e.g., foot leaves or fails to leave the ground) that consider successive FOG episodes separately.
